# MicroRNAs in ovarian function and disorders

**DOI:** 10.1186/s13048-015-0162-2

**Published:** 2015-08-01

**Authors:** Ying Li, Ying Fang, Ying Liu, Xiaokui Yang

**Affiliations:** Department of Human Reproductive Medicine, Beijing Obstetrics and Gynecology Hospital, Capital Medical University, Beijing, 100026 China; Department of Reproduction Regulation, Beijing Obstetrics and Gynecology Hospital, Capital Medical University, Beijing, 100026 China

**Keywords:** microRNA, Ovary, Follicle, Ovarian disorder

## Abstract

MicroRNAs (miRNAs) are endogenous, small, noncoding single-stranded RNA molecules approximately 22 nucleotides in length. miRNAs are involved in the post-transcriptional regulation of various important cellular physiological and pathological processes, including cell proliferation, differentiation, apoptosis, and hormone biosynthesis and secretion. Ovarian follicles are the key functional units of female reproduction, and the development of these follicles is a complex and precise process accompanied by oocyte maturation as well as surrounding granulosa cell proliferation and differentiation. Numerous miRNAs expressed in the ovary regulate ovarian follicle growth, atresia, ovulation and steroidogenesis and play an important role in ovarian disorders. This review considers recent advances in the identification of miRNAs involved in the regulation of ovarian function as well as the possible influence of miRNAs on ovarian-derived disorders, such as ovarian cancer, polycystic ovarian syndrome and premature ovarian failure. An improved understanding of the regulation of ovarian function by miRNAs may shed light on new strategies for ovarian biology and ovarian disorders.

**Electronic supplementary material:**

The online version of this article (doi:10.1186/s13048-015-0162-2) contains supplementary material, which is available to authorized users.

Discovered in 1993 by Ambros and colleagues, microRNAs (miRNAs) are endogenous, small, noncoding single-stranded RNA molecules 22–24 nucleotides (nt) in length [1]. Primary miRNA transcripts (pri-miRNAs) are several kilobases long and undergo substantial processing in the nucleus, resulting in the generation of a 70- to 90-nt stem-loop precursor miRNA (pre-miRNA). Pre-miRNAs undergo substantial processing by Dicer, a double-stranded miRNA duplex, to form mature miRNAs. Mature miRNAs play pivotal roles in regulating translation by binding to the 3′-untranslated regions (3′-UTRs) of their target mRNAs [1–4]. Exosome-mediated miRNA transfer is a major mechanism of genetic exchange between cells [5]. Kosaka et al. reported that circulatory miRNAs are released with exosomes, including miRNAs, mRNAs and proteins, and are subsequently transferred to recipients to resume their functions [6].

miRNAs are involved in the regulation of various important cellular physiological and pathological processes, including cell proliferation, differentiation, apoptosis, and hormone biosynthesis and secretion [5, 7]. The human ovary contains approximately 10,000 primordial follicles at birth that are prepared for the long duration of the fertile period. Folliculogenesis and steroidogenesis are complex processes involving intraovarian gene expression, signaling pathways, and endocrine and paracrine factors [8, 9]. The phosphorylation and tensin homolog deleted on chromosome ten/phosphatidylinositol-3 kinase/protein kinase B (PTEN/PI3K/Akt) signaling pathway contributes to oocyte proliferation, survival, migration and metabolism [10]. Wingless-type MMTV integration site family member 4 (WNT4) is required for antral follicle development by regulating granulosa cell functions [11], the transforming growth factor β (TGF-β) superfamily members (including GDF9, growth differentiation factor 9 and BMP15, bone morphogenetic protein 15) are major regulators of follicle development [12], and miRNAs are involved in granulosa cell proliferation and apoptosis [13].

Here, we review recent findings regarding miRNA expression profiles in the ovary and the potential roles of miRNA in ovarian function and ovarian disorders. We believe that a better understanding of ovarian miRNA function will lead to a new era of female reproductive health.

## miRNA profiles in the ovary

miRNAs are expressed in the ovary and are involved in the regulation of mammalian reproduction. miRNA expression profiles have been identified in numerous species (Additional file 1: Table S1), including human, mouse, bovine, sheep, chicken, fish, swine and equine species [14–21]. Timoneda et al. performed a systematic study of porcine miRNA expression and found that let-7a, miR-25 and miR-106a are preferentially expressed in the ovary [22]. Li et al. [21] evaluated the miRNA transcriptome in the adult porcine ovary and testis *via* deep sequencing technology. They reported that miR-21-5p, miR-143-3p and members of the let-7 family were the top unique miRNAs for both the ovary and testis and that these miRNAs play cellular housekeeping roles during ovarian and testicular development. miR-378, miR-1, miR-206, miR-379, miR-127, and miR-411 are downregulated in the ovary compared with the testis; by contrast, miR-10b, miR-26a, miR-21, miR-140, and miR-101 are upregulated in the ovary but not in the testis. Furthermore, most of the differentially expressed miRNAs located on the X chromosome (X-linked miRNAs) are significantly upregulated in the ovary compared with the testis and are co-expressed with X-linked miRNAs. Regardless of species, the let-7 family, miR-21, miR-99a, miR-125b, miR-126, miR-143, miR-145, and miR-199b are the most predominant miRNA populations in the ovary [23].

miRNA expression is organ specific and closely related to organ function, particularly in the ovary. The ovary contains oocytes and multiple somatic cell types such as granulosa cells, theca cells and cumulus cells. The expression and function of miRNAs are associated with different cell types (Additional file 2: Table S2). In total, 58 miRNAs are predominantly expressed in the bovine fetal ovary compared with somatic tissue. Among them, eight miRNAs (bta-miR-99a, bta-miR-10b, bta-miR-199a-3p, bta-miR-199a-5p, bta-miR-424, bta-miR-100, bta-miR-455, and bta-miR-214) are expressed at a 10-fold greater level in the fetal bovine ovary compared with somatic tissue pools. Further analyses indicate that bta-miR-424 and bta-miR-10b are highly abundant in germinal vesicle (GV) oocytes [20]. Such expression patterns indicate that these miRNAs are maternally inherited and may potentially be involved in the maternal transcript turnover during zygotic gene activation. Various miRNAs are involved in oocyte maturation, such as miR-2, miR-7, miR-184, miR-100, miR-9b, let-7, miR-79, miR-133, miR-275 and miR-252 [24–26]. In different stages of the oocytes, miRNA expression has been shown to differ. miR-2 and miR-133 are considerably more abundant in the first metaphase (MI) compared with the GV stage, and they both inhibit cyclin B translation by downregulating the 3′-UTRs of the crab cyclin B gene [25]. Some miRNAs, such as the let-7 family, play a housekeeping role in the ovary, independent of species [21, 27]. miRNA in granulosa cells is expressed differentially in the follicular and luteal stages. miR-503 has been found to be downregulated during the FSH-responsive follicular development stage and luteinization and upregulated during the later stage before ovulation [28].

In characterizing the miRNA profile in human cumulus granulosa cells (CGCs), Xu et al. demonstrated that the let-7 family was the most abundant miRNA in these cells in both polycystic ovarian syndrome (PCOS) patients and normal cycling women [27]. Compared with normal cycling women, miR-10a-5p, miR-1307-3p, miR-423-5p, miR-1273 g-3p, miR-199a-3p, miR-185-5p, and miR-483-5p are upregulated in the CGCs of PCOS patients, and miR-483-5p suppresses Notch3 and mitogen-activated protein kinase 3 (MAPK3) expression in human CGCs by directly binding the 3′-UTRs of Notch3 and MAPK3 mRNA [27].

Bioinformatics and Gene Ontology analysis revealed that the target genes of these predominantly expressed miRNAs in the ovary are involved in cell cycle regulation; cellular growth, proliferation and apoptosis; endocrine system disorders; and ovarian functions [29]. In addition, a recent study demonstrated that miR-143 inhibited primordial follicle formation by reducing the expression of cyclin-dependent kinases (CDKs) 4 and 6 and cyclins B1, D2, and E2 in pregranulosa cells [30]. Furthermore, miR-181a inhibits mouse ovarian granulosa cell proliferation by targeting activin receptor IIA [31], and miR-26b promotes ovarian granulosa cell apoptosis by targeting the ataxia telangiectasia mutated (ATM) gene during follicular atresia [32]. Finally, miR-132 and miR-212 expression are associated with hormonal regulation in ovulation and luteinization [33].

## miRNAs and ovarian function

### miRNAs and ovarian follicle development

miRNAs are involved in the entire process of ovarian follicle development, including follicle growth, atresia and ovulation. In each stage of follicle development, different growth factors contribute to stage-specific functions in different cell types [8, 9]. miRNAs also play an important role in the regulation of follicular development. McBride et al. [19] identified miRNA expression profiles at different stages of follicle development, including small follicles (1.5–3.5 mm), medium follicles (4.0–5.5 mm), pre-ovulatory follicles, early corpora lutea, late corpora lutea, and corpus albicans. miR-21, miR-125b, let-7a and let-7b are the most abundantly expressed miRNAs across the different development stages. miR-199a-3p, miR-145 and miR-31 are over-expressed at the follicular stage and exhibit a marked decrease in the follicular-luteal transition. By contrast, miR-503, miR-21 and miR-142-3p are generally expressed at lower levels during the follicular stages and exhibit significant increases in luteal tissues [19]. Zhang et al. [31] reported that miR-181a was reduced in preantral and antral follicles of mice compared with primary follicles. miR-181a suppressed activin receptor IIA (acvr2a) expression and decreased the phosphorylation of the activin intracellular signal transducer mothers against decapentaplegic homolog 2 (Smad2) in mice granulosa cells, leading to regulation of granulosa cell proliferation and ovarian follicle development [31].

Folliculogenesis begins with the breakdown of germ cell clusters and the formation of primordial follicles. Zhang et al. revealed that miR-143 was expressed in pregranulosa cells using *in situ* hybridization. miR-143 inhibits the formation of primordial follicles by suppressing pregranulosa cell proliferation and downregulating the expression of genes related to the cell cycle, including cyclin D2, CDK4 and CDK6 [30]. During folliculogenesis, more than 99 % of ovarian follicles undergo atresia, and the roles of miRNAs in regulating follicle development and atresia were recently elucidated. Differentially expressed miRNAs were constructed for healthy, early atretic, and progressively atretic follicles [32]. Hsa-miR-936, P-miR-1281, hsa-miR-26b, mmu-miR-1224, hsa-miR-10b, P-miR-466 g-b, P-miR-1275, hsa-miR-574-5p, R-miR-26b, hsa-miR-149*, hsa-miR-1275, and hsa-miR-99a are upregulated during follicle atresia, whereas R-let-7a, hsa-let-7i, hsa-miR-92b, hsa-miR-92a, P-miR-923, hsa-miR-1979, R-miR-739, hsa-miR-1308, hsa-miR-1826, P-miR-1826, and ssc-miR-184 are downregulated during this process. miR-26b, which is upregulated during follicular atresia, increases DNA breaks and promotes granulosa cell apoptosis by directly targeting ATM.

Follicle atresia is triggered by granulosa cells apoptosis [34, 35]. miRNAs are involved in granulosa cell apoptosis. miR-34s induces cell apoptosis and growth arrest through the activation of p53 and the cyclin-dependent kinase inhibitor p21 [36, 37]. Tu et al. reported that miR-34a promoted granulosa cell apoptosis in pig ovarian follicles by targeting the inhibin beta B (INHBB) gene [38]. Carletti et al. reported that miR-21 was highly induced by luteinizing hormone (LH) in murine granulosa cells and that the suppression of miR-21 activity *in vitro* caused granulosa cell apoptosis [39].

Advanced reports indicate that miRNA is associated with oocyte maturation. The oocyte initiates meiosis at the beginning of DNA synthesis and remains in MI phase until the resumption of meiosis. Before ovulation, oocytes become secondary oocytes after completing the first meiosis and stop at metaphase II (MII) of meiosis until fertilization [26, 40]. Xiao *et al.* [26] reported that transgelin 2 (TAGLN2), which encodes an actin protein, participates in ovarian development and maturation. In addition, miR-133b regulates oocyte maturation through its potential target TAGLN2 at both the transcription and translation levels.

Dicer is a ribonuclease that is required for the synthesis and processing of mature functional miRNAs. Dicer is expressed in both oocytes and granulosa cells of the mouse ovarian follicle [19]. The role of Dicer in pre-ovulatory follicle development and ovulation has also been elucidated. Lei et al. [28] reported that conditional inactivation of Dicer1 in follicular granulosa cells led to increased primordial follicle pool endowment, accelerated early follicle recruitment and an increase in degenerate follicles in Dicer conditional knockout (cKO) ovaries. Dicer1 regulates follicle development by downregulating miR-503, an ovary-specific miRNA, as well as miR-503 target genes, such as anti-Müllerian hormone (AMH); inhibin beta A subunit (INHBA); cytochrome P450, family 17, subfamily a, polypeptide 1 (Cyp17a1); cytochrome P450, family 19, subfamily a, polypeptide 1 (Cyp19a1); zona pellucida glycoproteins (ZPs); growth differentiation factor 9 (GDF9) and bone morphogenetic protein 15 (BMP15). Dicer1 inactivation in female mice causes abnormal follicular morphology and infertility [28, 40, 41]. These studies demonstrate that Dicer plays important roles in follicle growth and oocyte maturation.

Multiple factors are involved in follicle development, such as the TGF-β superfamily members [42, 43], Ligand stimulation of type I (also referred to as activin receptor-like kinases (ALKs)) and Smads [44–46]. miRNA regulates follicle development by affecting these factors. A recent study revealed that miR-224 expression is regulated by the TGF-β/Smad pathway. miR-224 overexpression enhances TGF-β1-induced granulosa cell proliferation by targeting Smad4, which is a key regulator involved in ovarian follicle growth and female fertility, whereas inhibition of endogenous miR-224 partially suppresses TGF-β1-induced granulosa cell proliferation, indicating an important biological role of miR-224 in regulating gene expression during folliculogenesis [47].

miRNAs also influence ovulation indirectly. Hasuwa et al. examined the role of miR-200b and miR-429 in anovulation and infertility in female mice [48]. miR-200b and miR-429 suppressed the expression of zinc-finger E-box binding homeobox 1 (ZEB1) in the pituitary gland where these miRNAs are highly expressed; moreover, miR-200b and miR-429 inactivation restrained LH biosynthesis, suggesting that miR-200b and miR-429 support ovulation by indirectly functioning in the hypothalamus-pituitary-ovarian axis.

### miRNAs and ovarian steroidogenesis

Folliculogenesis is a highly dynamic process that is closely associated with alterations in circulating hormone levels. Given the indispensable role of miRNAs in ovarian follicle development and female fertility, the relationships between ovarian hormones and miRNAs have recently been investigated. A study on granulosa/cumulus cells reported the expression of several miRNAs (miR-23a, miR-23b, miR-542-3p, miR-211, and miR-17-5p) in ovarian somatic cells, and the authors speculated that miRNA target genes, including cyclooxygenase-2, steroidogenic acute regulatory protein (StAR), and CYP-19A1 (aromatase), regulate granulosa/theca cells proliferation, differentiation and steroid biosynthesis [43].

miRNAs regulate ovarian steroid hormones by targeting hormone receptors as well as affecting hormone biosynthesis and release. For example, estradiol (E2) plays an important role in ovarian follicle development, and the production of E2 is tightly controlled by aromatase. Xu et al. reported that aromatase expression and estradiol synthesis in granulosa cells are post-transcriptionally downregulated by miR-378 and that miR-378 affects estradiol synthesis by binding the 3′-UTR of the aromatase coding sequence [49]. Conversely, miR-133b stimulates ovarian estradiol synthesis by targeting Foxl2, which mediates the transcriptional repression of StAR and CYP19A1 to promote estradiol biosynthesis [50]. miRNAs not only regulate estradiol synthesis but also estradiol release. Estradiol release is promoted by miR-383 in ovarian granulosa cells, and miR-383 inhibits RNA binding motif single-stranded interacting protein 1 (RBMS1) by altering its mRNA stability, leading to the inactivation of c-Myc and steroidogenesis in granulosa cells [51]. Finally, miR-423-5p and miR-378 regulate estradiol synthesis by targeting CYP19A1 mRNA and repressing CYP19A1 protein content and enzyme activity in newborn piglets [52].

Conversely, the miRNA expression profile is profoundly influenced by circulating hormones. miR-132 and miR-212 expression are increased following human chorionic gonadotropin (HCG) induction [33], and the expression of 31 microRNAs is altered after follicle-stimulating hormone (FSH) treatment. Specifically, miR-29a and miR-30d expression is downregulated in the short term but upregulated in the long term following FSH induction [53].

Sirotkin et al.[54] first demonstrated that miRNAs control reproductive functions, resulting in enhanced or inhibited release of ovarian progestagen and androgen. Thirty-six miRNAs, including let-7b, let-7c, miR-15a, miR-17-3p, miR-96, miR-92, miR-108, miR-133b, miR-134, miR-135, and miR-146, inhibited progesterone release, whereas 16 miRNAs (miR-16, miR-24, miR-25, miR-122, miR-145, miR-182, miR-18, miR-125a, miR-147, miR-32, miR-103, miR-143, miR-150, miR-152, miR-153 and miR-191) promoted progesterone release in granulosa cells. In addition, let-7a, let-7b, let-7c, miR-16, miR-17-3p, miR-24, miR-25, miR-26a, mir-108, and mir-122 inhibited testosterone release.

Moreover, pathological factors significantly influence miRNA expression in the mammalian ovary. Bisphenol-A (BPA) is an environmentally ubiquitous endocrine system-disrupting chemical. Veiga-Lopez et al. studied the effect of BPA on mRNA and miRNA expression and reported that BPA increased CYP19 and 5α-reductase mRNA expression, disrupted the sheep ovarian transcriptome and altered the fetal ovarian miRNA expression profile [55]. In addition, ovarian miRNA expression, including miR-497 and miR-15b, is influenced by prenatal testosterone treatment [56].

### miRNAs and ovarian disorders

Recent studies have reported that differential expression and dysregulation of miRNAs are associated with ovarian diseases, such as ovarian cancer, PCOS and premature ovarian failure (POF) [13, 57].

### miRNAs and ovarian cancer

Ovarian cancer is the most lethal gynecological malignancy. Recently, multiple studies have profiled miRNAs in ovarian cancer compared with normal tissues to identify differentially expressed miRNAs [58–61]. Iorio et al. [58] were the first to compare genome-wide miRNA expression profiles from both ovarian cancer tissues and normal ovary tissues and reported that miRNA expression was differentially regulated in the two groups; miR-200a, miR-141, miR-200c, and miR-200b were overexpressed in ovarian cancer, and miR-199a, miR-140, miR-145, and miR-125b1 were the most downregulated miRNAs. These four downregulated miRNAs had a common target oncosuppressor, namely, BRCA1-associated protein (BAP1). Shapira and his colleagues [60] collected presurgical plasma samples from women with confirmed serous epithelial ovarian cancer, benign neoplasms and no known pelvic mass to assess miRNA profiles. Twenty-two miRNAs were differentially expressed between healthy controls and the ovarian cancer group, whereas a six-miRNA-profile subset (miR-106b, miR-126, miR-150, miR-17, miR-20a, and miR-92a) could distinguish between benign and ovarian cancer patients.

miRNAs play significant roles in the early diagnosis, prognosis and chemotherapy sensitivity of ovarian cancer. A recent study related to stage I ovarian tumors revealed that miR-30a and miR-30a* are markers of clear-cell tumors, whereas miR-192 and miR-194 are markers of mucinous tumors [62]. Langhe et al. [63] reported that 4 miRNAs (let-7i-5p, miR-122, miR-152-5p and miR-25-3p) are significantly downregulated in ovarian cancer patients. The target genes of these differentially expressed miRNAs are involved in WNT signaling, AKT/mTOR and TLR-4/MyD88, which play roles in ovarian carcinogenesis and chemoresistance. These results indicate the roles of let-7i-5p, miR-122, miR-152-5p and miR-25-3p as diagnostic biomarkers in ovarian cancer.

In a prognostic study, Merritt investigated Dicer messenger RNA (mRNA) and Drosha levels in invasive epithelial ovarian cancer patients and compared the results with clinical outcomes. Low Dicer expression was significantly associated with advanced tumor stage, and low Drosha expression was associated with suboptimal surgical cytoreduction [64]. Another study by Marchini [65] et al. confirmed that miR-200c downregulation is associated with overall and progression-free survival (PFS) independent of clinical covariates in stage I epithelial ovarian cancer. Further research by Park [66] identified the pivotal role of the miR-200 family in the epithelial-to-mesenchymal transition (EMT), which is a decisive step toward tumor cell invasion and metastasis and is positively correlated with poor patient prognosis. miR-200 suppressed the EMT by directly downregulating the expression of the E-cadherin transcriptional repressors ZEB1 and ZEB2 (SMAD-interacting protein 1, SIP1), thus reducing E-cadherin expression and promoting the EMT [66]. In studies of the serous subtype of epithelial ovarian cancer, miR-506 was found to be a robust EMT inhibitor through direct targeting of the E-cad repressor SNAI2 [67], the vimentin gene (VIM) and N-cad gene (CDH2) [68], suggesting that miR-506 inhibits multiple targets in the EMT network and is associated with good prognosis in epithelial ovarian cancer. Regarding the therapeutic role of miRNAs in ovarian cancer, Gu et al. [69] reported that three miRNAs (hsa-miR-146a, hsa-miR-148a and hsa-miR-545) are predominantly expressed in patients with wild-type BRCA1/2 ovarian cancers who may benefit from platinum-based chemotherapy. These miRNAs target BRCA1/2, which is the key gene involved in the DNA damage response and DNA repair processes, leading to the increasing sensitivity of cancer cells to chemotherapy [69].

### miRNAs and PCOS

PCOS is a multifactorial endocrine disorder affecting approximately 5–10 % of all women of reproductive age [70–72]. Hossain et al. [73] established a rat PCOS model *via* dihydrotestosterone (DHT) induction to investigate PCOS-associated ovarian miRNA expression profiles and found that the differential expression of regulatory miRNAs is associated with PCOS pathogenesis in rat ovaries. This study showed that 25 miRNAs, which were designated as ovarian miRNAs, were highly and differently expressed in the ovary in PCOS and normal rats. Most of the miRNAs in the ovary that promote the cystic conditions are localized in the follicular theca cells of DHT-treated ovaries. Further research explored the dysregulated molecular pathways related to altered miRNAs in PCOS rat ovaries. miR-222 was confirmed to be expressed in theca cells by *in situ* localization, and the expression was reported to be repressed by androgens, which regulate cell proliferation by targeting P27/kip1. In addition, miR-222 overexpression was associated with reduced ERα protein and signaling as well as expression of the ERα target genes. The findings may offer new insights for PCOS pathogenesis research [73]. Sang et al. [74] assessed miRNA expression in human follicular fluid of PCOS patients and identified numerous miRNAs that play important roles in steroidogenesis. miR-132 and miR-320 are expressed at a significantly reduced level in the follicular fluid of polycystic ovary patients compared with healthy controls. In addition, miR-132, miR-320, miR-520c-3p, miR-24 and miR-222 regulate estradiol concentrations, and miR-24, miR-193b, and miR-483-5p regulate progesterone concentrations in PCOS patients.

PCOS is characterized by polycystic ovaries, hyperandrogenism, insulin resistance (IR) and chronic anovulation [75]. miRNAs are also involved in metabolic processes. For example, miR-93 is overexpressed in PCOS and is associated with decreased GLUT4 and increased IR [76]. Additionally, miRNA-21, miRNA-27b, miRNA-103 and miRNA-155 play important roles in metabolic processes and are influenced by obesity and circulating androgen concentrations in PCOS patients [77].

### miRNAs and premature ovarian failure (POF)

POF is an ovarian disorder of multifactorial origin that is defined as the occurrence of amenorrhea, hypergonadotropism and hypoestrogenism in women aged younger than 40 years [78]. Recent studies based on samples from both plasma and ovarian tissues have identified miRNAs involved in POF development. Dang et al. reported reduced miR-22-3p plasma levels in POF of Han Chinese patients compared with control women. In addition, decreased miR-22-3p expression was correlated with the diminished ovarian reserve [79]. Our previous studies identified the differentially expressed miRNAs in plasma between POF and normal cycling women and the roles of miRNAs in regulating many signaling pathways [13]. miR-23a is upregulated in the plasma of POF patients, and miR-23a overexpression decreases XIAP and caspase-3 levels and increases apoptosis in human granulosa cells. These results indicate that miR-23a potentially induces granulosa cell apoptosis by inhibiting XIAP expression both at the mRNA and protein levels *in vitro* [13]. In addition, Kuang et al. [80] identified a total of 63 upregulated and 20 downregulated miRNAs in ovarian tissue samples from 4-vinylcyclohexene diepoxide (VCD)-induced rat POF models compared with samples from normal rats. Further studies confirmed that miR-29a and miR-144 are downregulated in POF tissues and potentially regulate prostaglandin biosynthesis by targeting PLA2G4A, whereas various upregulated miRNAs, including miR-27b, miR-190, miR-151 and miR-672, are involved in the apoptotic process and hormone stimulation [80].

Recent studies indicate that miRNA single-nucleotide polymorphisms (SNPs) are associated with disease susceptibility. A study related to miRNA polymorphism analysis identified the association between combined genotypes and haplotypes of miR-146aC>G, miR-196a2T>C, and miR-499A>G and POF in Korean women; the results indicate that the transcriptional aberration of miR-146a and miR-196a2 induced by miRNA SNPs is potentially involved in POF development [81].

## Conclusions and future directions

miRNAs are post-transcriptional regulators in both physiological and pathological processes. A single miRNA may target several mRNAs, and a single mRNA may be regulated by multiple miRNAs. Many miRNAs are expressed in the ovary and are involved in ovarian follicle development, atresia, ovulation and ovarian steroidogenesis by targeting specific genes and regulating various signaling pathways. miRNAs also play important roles in ovarian diseases. However, studies on miRNAs in the ovary have mainly focused on expression profiles rather than their regulation and function networks. Identifying miRNAs that are specific to different reproductive organs will help guide researchers to better understand the underlying mechanisms of reproductive disorders. In addition, identifying upstream or additional regulators of miRNAs, their target genes and their roles in the related signaling pathways will further shed light on the importance of specific miRNAs for both the development and function of the ovary, paving the way for new therapeutic strategies by controlling the key factors in the regulatory networks. Furthermore, recent studies have demonstrated that SNPs located in miRNA genes or miRNA binding sites potentially modify miRNA regulation, thus affecting phenotypes and disease susceptibility. Therefore, a combination of miRNA expression profiles with genome-wide SNP genotyping might help distinguish among potential disease-related biomarkers. A better understanding of the regulation of ovarian function by miRNAs may offer a theoretical foundation for ovarian diseases.

## Additional files

Additional file 1: Table S1. Ovarian microRNAs in different species. (DOCX 16 kb) Additional file 2: Table S2. miRNAs expressed in GCs and oocytes. (DOCX 40 kb)

## Abbreviations

3'UTR: 3' untranslated regions
Akt: Protein kinase B
ALK: Activin receptor-like kinase
AMH: Anti-Müllerian hormone
ATM: Ataxia telangiectasia mutated
BMP: Bone morphogenetic protein
BRCA1: Breast-cancer susceptibility genes 1
CDK: Cyclin-dependent kinase
CGC: Cumulus granulosa cells
COX: Cyclooxygenase
EMT: Epithelial-to-mesenchymal transition
FSH: Follicle stimulating hormone
GDF9: Growth differentiation factor 9
GV: Germinal vesicle
HCG: Human chorionic gonadotropin
INHBB: Inhibin beta B
LH: Luteinizing hormone
MI: First metaphase
MII: Second metaphase
miRNA: microRNA
PCNA: Proliferating cell nuclear antigen
PCOS: Polycystic ovarian syndrome
PFS: Progression-free survival
PI3K: Phosphatidylinositol-3 kinase
POF: Premature ovarian failure
pre: Precursor
pri: Primary
PTEN: Phosphorylation and tensin homolog deleted on chromosome ten
RBMS: RNA binding motif single stranded interacting protein
Smad: Signal transducer mothers against decapentaplegic homolog
StAR: Steroidogenic acute regulatory protein
TAGLN2: Transgelin 2
TGF-β: Transforming growth factor β
TLR-4: Toll-like receptor 4
WNT: Wingless-type MMTV integration site family member
XIAP: X-linked inhibitor of apoptosis protein
ZEB: Zinc-finger Ebox Binding Homeobox
ZPs: Zona pellucida glycoproteins
