# Supplementary material for: MicroRNAs in ovarian function and disorders
Source: J Ovarian Res. 2015 Aug 1;8:51. doi: 10.1186/s13048-015-0162-2 (PMC4522283; doi:10.1186/s13048-015-0162-2)
Supplement: Additional file 1: Table S1. — Ovarian microRNAs in different species. (DOCX 16 kb) [file 13048_2015_162_MOESM1_ESM.docx]

| **Additional file 1: Table S1 Ovarian microRNAs in different species** | | |
| --- | --- | --- |
| **Species** | **microRNAs in Ovary** | **Reference(s)** |
| **Human** | *let-7d, let-7e, let-7g*  *has-mir-106b; has-mir-142-3p; has-mir-146a* | [17] |
| **Mouse** | *mir-ov2, mir-ov3,mir -ov4*  *mir-ov6-1, mir-ov6-2*  *mir-ov9-1, mir-ov9-2, mir-ov9-3*  *mir-ov11; mir-503* | [12] |
| **Bovine** | *let-7a, let-7b, let-7c, let-7d, let-7f*  *bta-mir-10a, bta-mir-10b*  *bta-mir-99a; bta-mir-100;bta-mir-103;bta-mir-107*  *mir-125a, mir-125b*  *bta-mir-199a-3p, bta-mir-199a-5p*  *bta-mir-214; bta-mir-424; bta-mir-455* | [17, 18] |
| **Sheep** | *let-7a, let-7c, let-7d, let-7g, let-7e*  *oar-mir-127; oar-mir-329b-3p*  *oar-mir-376c-3p; oar-mir-379-5p*  *oar-mir-411a-3p,oar-mir-411b-5p, oar-mir-411b-3p* | [13, 17] |
| **Chicken** | *gga-mir-let-7a, gga-mir-let-7b, gga-mir-let-7c, gga-mir-let-7j, gga-mir-let-7f, gga-mir-let-7k*  *gga-mir-10a; gga-mir-21; gga-mir-30e*  *gga-mir-101; gga-mir-146c; gga-mir-148a* | [14] |
| **Rainbow Trout** | *mir-101; mir-126; mir-196*  *mir-202, mir-301, mir-338* | [15] |
| **Porcine** | *let-7 family*  *mir-21-5p; mir-25; mir-106a*  *mir-136-3p; mir-143-3p; mir-151-3p;mir-224-5p* | [19] |
| **Equine** | *mir-10a; mir-16;mir-18a;mir-20a ,*  *mir-26b;mir-28-3p*  *mir-30c, mir-30d* | [16] |
