# Supplementary material for: MicroRNAs in ovarian function and disorders
Source: J Ovarian Res. 2015 Aug 1;8:51. doi: 10.1186/s13048-015-0162-2 (PMC4522283; doi:10.1186/s13048-015-0162-2)
Supplement: Additional file 2: Table S2. — miRNAs expressed in GCs and oocytes. (DOCX 40 kb) [file 13048_2015_162_MOESM2_ESM.docx]

| **Additional file 2: Table S2 miRNAs expressed in GCs and oocytes** | | | | | |
| --- | --- | --- | --- | --- | --- |
| **miRNAs** | **Species** | **Regulation** | **Target genes** | **Functions** | **Ref^a^** |
| **Granulosa cells** | | | | | |
| miR-503 | Mouse | Stimulation by gonadotrophins; Down-regulated during FSH-responsive follicular development stage and luteinization; Up-regulated during later stage before ovulation | *ACTRIIa;ACTRIIb*  *FSHR;BCL2;*  *CCND2* | GC proliferation and luteinization | [1] |
| miR-21; miR-132  miR-212; miR-224 | Equine | Up-regulated by hCG | *PTEN;RASA1*  *SMAD4* | Regulation of steroidogenesis  and ovulation | [2] |
| miR-10a;miR-105  miR-182;miR-15a | Human  Rat | miR-182:  Up-regulated by cAMP agonist | *CyclinB1;TdT*  *Caspase-3;PCNA* | Involve in GC  proliferation and apoptosis | [3]; [4] |
| miR-224 | Mouse | Up-regulated by TGF-β1/SMAD pathway | *SMAD4* | TGF-β1-mediated GC growth and E2 production | [5] |
| miR-23a | Human | Capase-3 dependent apoptosis pathway | *XIAP;Caspase-3* | Pro-apoptotic role | [6] |
| miR-383 | Mouse | Down-regulated by TGF-β1 and transcription factor steroidogenic factor-1 (SF-1) | *RBMS1* | Promote steroidogenesis in GC | [5]; [7] |
| miR-320 | Mouse | Down-regulated by TGF-β1, FSH, and pregnant mare serum gonadotropin (PMSG) | *E2F1*  *SF-1* | Inhibit E2 synthesis and GC proliferation;  Promotion of T and P synthesis | [5]  [8] |
| miR-29a  miR-30d | Rat | Down-regulation after FSH treatment for 12 hours and  up-regulation after FSH treatment for 48 hours | *COL4A1;BMF*  *RNF2;EED* | Involvement in FSH-induced progesterone production | [9] |
| miR-145 | Mouse | - | *ACVRIB* | Suppress GC proliferation | [10] |
| miR-21 | Mouse  Human | Up-regulated by hCG/LH | *COL4A1* | Anti-apoptosis role in GC;  Regulate COL4A1 synthesis | [11]  [12] |
| miR-132  miR-214 | Rat | down-regulated by cAMP agonist(miR-132);  up-regulated by cAMP agonist(miR-214) | *SREBP-1c LDLR* | Lipid metabolism  /steroidogenesis in rat GC | [4] |
| miR-181a | Mouse | - | *ACVRIIA* | Supress GC proliferation | [13] |
| miR-125b | Human  Mouse | induced by dihydrotestoster-1 and testosterone | *BAK;BAX*  *BMF;TP53* | Suppression of proapoptotic protein expression in GCs | [14] |
| let-7 family; miR-21;miR-143;  miR-125b; | Mouse | Housekeeping  Regulated by FSH treatment; | - | Follicle development | [15] |
| miR-26b | Pig | - | *ATM* | Pro-apoptotic role | [16] |
| **Oocytes** | | | | | |
| miR-184;miR-10a  miR-100 | Human | - | *SMARCA5*  *NCOR2;HOXA1* | Oocyte reprogramming;  Repression nuclear receptors;  Regulation of oocyte-specific  gene expression | [17-20] |
| miR-224 | Mouse | Up-regulated by TGF-β1 and EGF | *PTX3* | Cumulus expansion in EGF-stimulated COCs | [21] |
| miR-205 miR-150  miR-122;miR-96,  miR-146a; iR-146b-5p | Bovine | Dynamic degradation during  oocyte maturation | - | Oocyte maturation | [22] |
| miR-335-5p | Mouse | At high level mainly during the meiotic  Maturation period; Decreased significantly  shortly after fertilization | *Daam1;ERK1/2*  *Mitogen-activated*  *protein kinase pathway* | Oocyte meiosis;  Cytoskeleton dynamics;  Spindle formation | [23] |
| miR-20a;miR-15a  miR-602 | Human | miR-20a,  miR-15a: dynamic changes during meiosis | miR-15a:  *BCL-2 family;CDC25A* | Regulation of  cell division and cell growth | [24] |
| let-7b;let-7c  miR-27a;miR-322 | Mouse | - | *IGFBP-2* | Regulation of oocyte meiotic competence | [25] |

^a^ The supplementary references are listed in Appendix S1.

**References:**

1. Lei, L., et al., The regulatory role of Dicer in folliculogenesis in mice. Mol Cell Endocrinol, 2010. **315**(1-2): 63-73.

2. Schauer, S.N., et al., Involvement of miRNAs in equine follicle development. Reproduction, 2013. **146**(3): 273-82.

3. Sirotkin, A.V., et al., Identification of microRNAs controlling human ovarian cell proliferation and apoptosis. J Cell Physiol, 2010. **223**(1): 49-56.

4. Hu, Z., et al., Hormonal regulation of microRNA expression in steroid producing cells of the ovary, testis and adrenal gland. PLoS One, 2013. **8**(10): e78040.

5. Yao, G., et al., MicroRNA-224 is involved in transforming growth factor-beta-mediated mouse granulosa cell proliferation and granulosa cell function by targeting Smad4. Mol Endocrinol, 2010. **24**(3): 540-51.

6. Yang, X., et al., Differentially expressed plasma microRNAs in premature ovarian failure patients and the potential regulatory function of mir-23a in granulosa cell apoptosis. Reproduction, 2012. **144**(2): 235-44.

7. Yin, M., et al., Transactivation of microRNA-383 by steroidogenic factor-1 promotes estradiol release from mouse ovarian granulosa cells by targeting RBMS1. Mol Endocrinol, 2012. **26**(7): 1129-43.

8. Yin, M., et al., Transactivation of micrornA-320 by microRNA-383 regulates granulosa cell functions by targeting E2F1 and SF-1 proteins. J Biol Chem, 2014. **289**(26): 18239-57.

9. Yao, N., et al., Follicle-stimulating hormone regulation of microRNA expression on progesterone production in cultured rat granulosa cells. Endocrine, 2010. **38**(2): 158-66.

10. Yan, G., et al., MicroRNA-145 suppresses mouse granulosa cell proliferation by targeting activin receptor IB. FEBS Lett, 2012. **586**(19): 3263-70.

11. Carletti, M.Z., S.D. Fiedler and L.K. Christenson, MicroRNA 21 blocks apoptosis in mouse periovulatory granulosa cells. Biol Reprod, 2010. **83**(2): 286-95.

12. Mase, Y., et al., MiR-21 is enriched in the RNA-induced silencing complex and targets COL4A1 in human granulosa cell lines. Reprod Sci, 2012. **19**(10): 1030-40.

13. Zhang, Q., et al., MicroRNA-181a suppresses mouse granulosa cell proliferation by targeting activin receptor IIA. PLoS One, 2013. **8**(3): e59667.

14. Sen, A., et al., Androgens regulate ovarian follicular development by increasing follicle stimulating hormone receptor and microRNA-125b expression. Proc Natl Acad Sci U S A, 2014. **111**(8): 3008-13.

15. Yao, N., et al., A network of miRNAs expressed in the ovary are regulated by FSH. Front Biosci (Landmark Ed), 2009. **14**: 3239-45.

16. Lin, F., et al., miR-26b promotes granulosa cell apoptosis by targeting ATM during follicular atresia in porcine ovary. PLoS One, 2012. **7**(6): e38640.

17. Assou, S., et al., MicroRNAs: new candidates for the regulation of the human cumulus-oocyte complex. Hum Reprod, 2013. **28**(11): 3038-49.

18. Assou, S., et al., A gene expression signature shared by human mature oocytes and embryonic stem cells. BMC Genomics, 2009. **10**: 10.

19. Wu, J., et al., MicroRNA-184 downregulates nuclear receptor corepressor 2 in mouse spermatogenesis. BMC Dev Biol, 2011. **11**: 64.

20. Lund, A.H., miR-10 in development and cancer. Cell Death Differ, 2010. **17**(2): 209-14.

21. Yao, G., et al., MicroRNA-224 is involved in the regulation of mouse cumulus expansion by targeting Ptx3. Mol Cell Endocrinol, 2014. **382**(1): 244-53.

22. Abd, E.N.W., et al., Expression analysis of regulatory microRNAs in bovine cumulus oocyte complex and preimplantation embryos. Zygote, 2013. **21**(1): 31-51.

23. Cui, X.S., et al., Involvement of microRNA-335-5p in cytoskeleton dynamics in mouse oocytes. Reprod Fertil Dev, 2013. **25**(4): 691-9.

24. Xu, Y.W., et al., Differentially expressed micoRNAs in human oocytes. J Assist Reprod Genet, 2011. **28**(6): 559-66.

25. Kim, Y.J., et al., MicroRNAs transfected into granulosa cells may regulate oocyte meiotic competence during in vitro maturation of mouse follicles. Hum Reprod, 2013. **28**(11): 3050-61.
